# Comparison of diagnostic yield of core-needle and fine-needle aspiration biopsies of thyroid lesions: Systematic review and meta-analysis

**DOI:** 10.1007/s00330-016-4356-9

**Published:** 2016-04-18

**Authors:** Kosma Wolinski, Adam Stangierski, Marek Ruchala

**Affiliations:** Department of Endocrinology, Metabolism and Internal Medicine, Poznan University of Medical Sciences, 60-355 Poznan, Przybyszewskiego 49 Str., Poznan, Poland

**Keywords:** Fine-needle aspiration biopsy, Core-needle biopsy, Thyroid· Thyroid lesions, Biopsy

## Abstract

**Objectives:**

Thyroid nodular disease is one of the most commonly observed medical conditions. Cytological evaluation of the specimens obtained with fine-needle aspiration biopsy (FNAB) is the most accurate tool for selecting nodules which should be further surgically removed. A major limitation of this method is the high occurrence of non-diagnostic results. This indicates the need for improvement of the thyroid biopsy technique. The aim of this meta-analysis was to compare the diagnostic value of thyroid core-needle biopsies (CNBs) and FNABs.

**Materials and methods:**

PubMed/MEDLINE, Cochrane Library, Scopus, Cinahl, Academic Search Complete, Web of Knowledge, PubMed Central, PubMed Central Canada and Clinical Key databases were searched. Risk ratios (RRs) of non-diagnostic results were meta-analysed using the random-effects model.

**Results:**

Eleven studies were included in the quantitative analysis. CNB yielded significantly more diagnostic results – the pooled risk ratio (RR) of gaining a non-diagnostic result was 0.27 (p<0.0001). For lesions with one previous non-diagnostic FNAB, RR was 0.22 (p<0.0001).

**Conclusions:**

CNB seems to be a valuable diagnostic technique yielding a higher proportion of diagnostic results than conventional FNAB. It is also significantly more effective in case of nodules with a prior non-diagnostic result of FNAB results than repeated FNABs.

***Key Points*:**

• *Core-needle biopsy yields a higher proportion of diagnostic results than fine-needle biopsy.*

• *Core-needle biopsies may decrease the amount of unnecessary thyroidectomies.*

• *Probability of gaining non-diagnostic result using core-needle biopsy is almost four times lower.*

## Introduction

Thyroid nodular disease (TND) is one of the most commonly observed medical conditions, affecting a large number of individuals, especially women, subpopulations in iodine-deficient regions, elderly people and patients with some specific clinical conditions. The prevalence of TND is high, affecting 10–70 % of the general population and malignancies are observed in 3–10 % of patients [1–5]. Cytological evaluation of the specimens obtained with fine-needle aspiration biopsy (FNAB) is the most accurate tool for selecting nodules which should be further surgically removed (malignancies, indeterminate follicular lesions) [6]. One of the major limitations of this method is a high occurrence of non-diagnostic results, falling in group I of the Bethesda Classification [7]. According to numerous studies, around 10–20 % of FNABs yield non-diagnostic results [8–10]. Most endocrinological societies recommend consideration of total thyroidectomy in cases of repeated FNABs with non-diagnostic results [11]. This may increase the number of unnecessary thyroidectomies and also delay the final diagnosis of thyroid cancer. This indicates the need for improvement of the thyroid biopsy technique or even searching for new tools which may decrease the prevalence of non-diagnostic results. Biopsy with the use of a core needle (CNB) is believed to be reliable improvement on FNAB, bringing high diagnostic yield [12, 13]. The aim of the current meta-analysis was to compare the diagnostic value of thyroid CNBs and FNABs.

## Materials and methods

### Study selection

PubMed/MEDLINE, Cochrane Library, Scopus, Cinahl, Academic Search Complete, Web of Knowledge, PubMed Central, PubMed Central Canada and Clinical Key databases from January 2001 up to December 2014 were searched in order to find all relevant, full-text journal articles written in English. We used the search term: ((“core-needle”) or (core and needle)) and thyroid. Articles comparing the percentage of diagnostic results of thyroid FNAB and CNB, performed with sonographic guidance, were included in the meta-analysis. According to the *Bethesda System for Reporting Thyroid Cytopathology* [14], categories II–VI are interpreted as diagnostic results. Samples classified as Bethesda category III and IV are inconclusive results in the context of differentiation between benign and malignant lesions but assessed as adequate for cytological assessment. We excluded studies about very particular groups of lesions (e.g. hyalinasing trabecular tumours, follicular tumours) and studies where FNAB or CNB was performed without ultrasound guidance. Studies without control groups, comparing results of FNAB with FNAB and CNAB performed simultaneously (without distinct data about the FNAB and CNAB results) were systematically reviewed.

Two researchers (K.W. and A.S.) searched all included databases independently and prepared a list of included studies. In case of discrepancies between lists, authors read questionable articles together.

### Quality assessment of the studies

All included studies were assessed using the Newcastle-Ottawa Scale [15]. Studies with a result of seven stars or more were included.

### Statistical analysis

All calculations were performed using Statistica v.10 with the medical package from Statsoft. Risk ratios (RRs) of non-diagnostic result were meta-analysed using the random-effects model. Publication bias was assessed using Kendall’s tau.

## Results

**Fig. 1:**
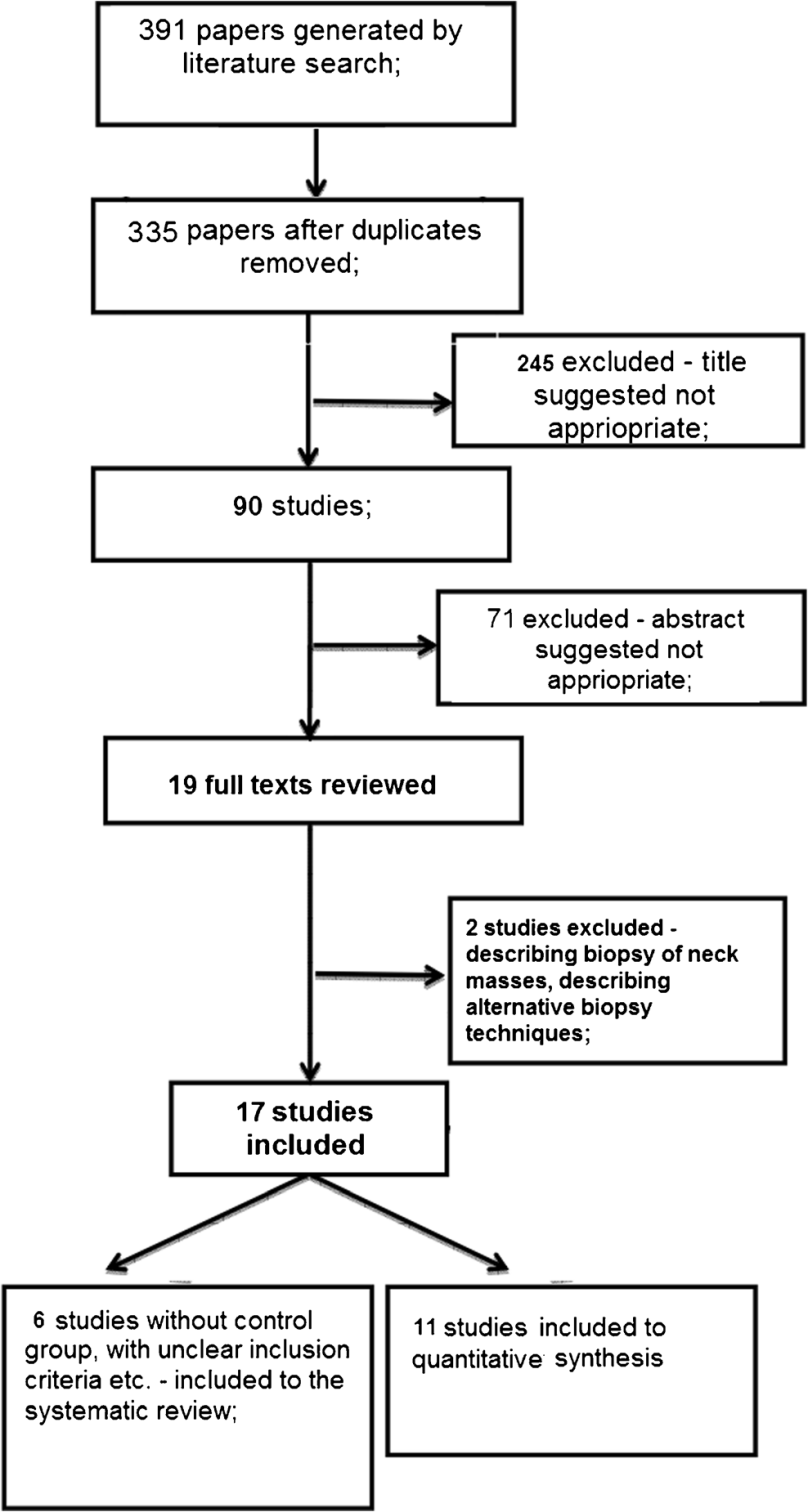
Flowchart showing the steps included in the literature search and selection

**Fig. 2:**
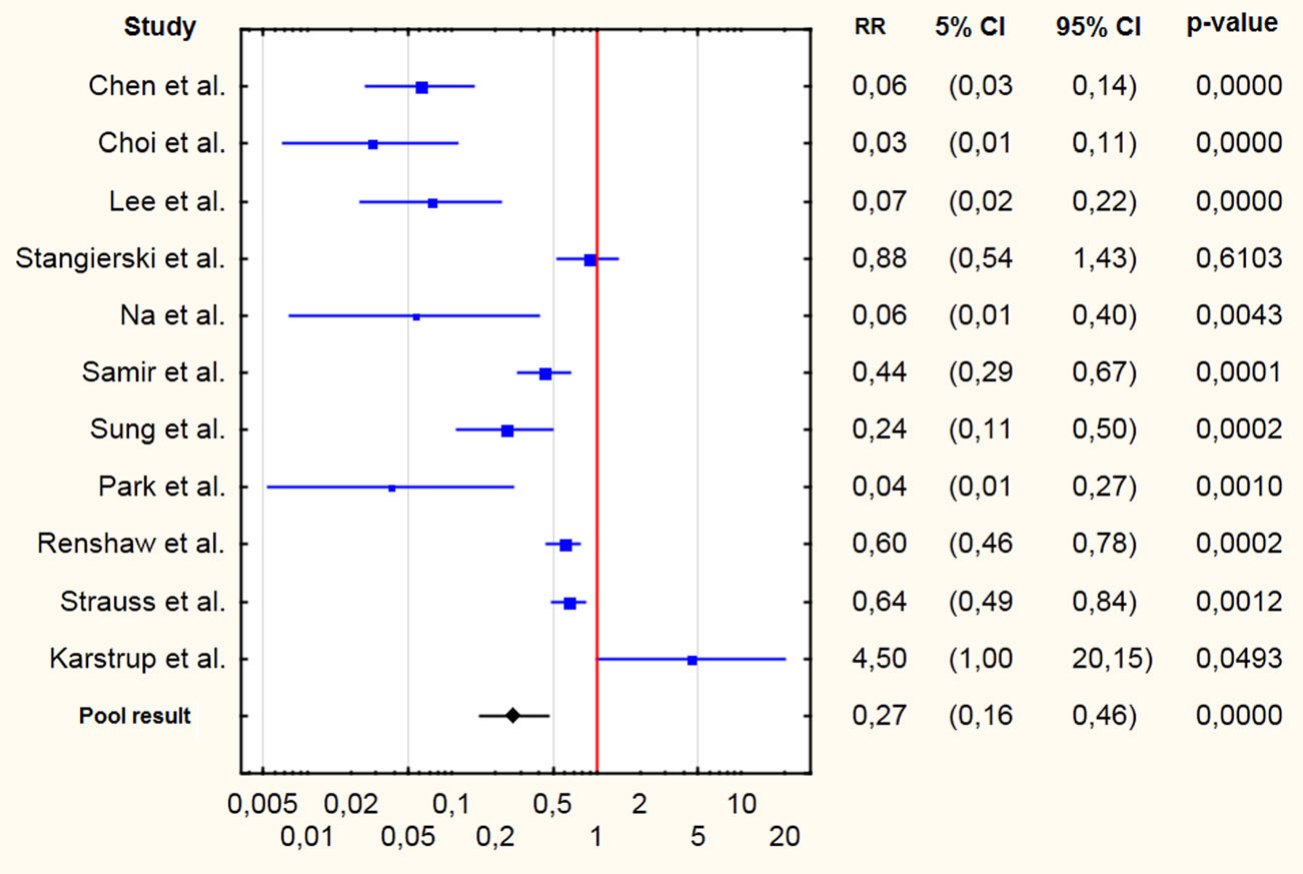
Forest plot showing individual and pooled risk ratios (RRs) of gaining non-diagnostic results with core-needle biopsy in comparison to fine-needle aspiration biopsy; with 95 % confidence intervals and p-values given in columns 2–4

The search results and steps of selection are shown in the flowchart (Fig. 1). Eleven studies were included to the meta-analysis – the basic data are shown in Table 1 [6, 16–25]. CNB yielded significantly a higher amount of diagnostic results. The forest plot is shown on Fig. 2. The pooled RR of non-diagnostic results was 0.27 with a 95 % confidence interval (CI) 0.16–0.46 (p<0.0001). There is no evidence for publication bias (Kendall’s tau = −0.24, two-tailed p-value = 0.31). There was evidence of significant heterogeneity (Q =85.3, df=10, i^2^=88.3 %, p<0.0001).

**Table 1:** Studies comparing the diagnostic efficacy of core-needle biopsy (CNB) and fine-needle aspiration biopsy (FNAB) in lesions with a previous non-diagnostic FNAB result

Author	Year	Country	Design	Needles	FNAB – diagn.	FNAB – ndg.	CNB – diagn.	CNB – ndg.
Chen et al. [16]	2014	USA	Retrospective; no specific selection criteria – FNAB and CNB interchangeably dependent on the preference of the radiologist	FN: 25–27 G; CN: 20 G, semi-automatic biopsy device	70	26	359	6
Choi et al. [17]	2014	South Korea	Retrospective; lesions with previous ndg.	FNA: 21–23 G; CN: 18 G; automatic biopsy gun used	108	72	178	2
Lee et al. [18]	2014	South Korea	Retrospective; lesions with previous ndg.	FN: no data; CN: 18 G; automatic biopsy gun used	260	129	122	3
Stangierski et al. [19]	2013	Poland	Prospective; lesions with previous ndg.	FN: 25 G; CN: 22 G	30	29	17	13
Na et al. [20]	2012	South Korea	Prospective; FNAB and CNB simultaneously; lesions with previous ndg.	FN: 25, 23 and 21 G; CN: 18 G; automatic biopsy gun used	46	18	63	1
Samir et al. [21]	2012	USA	Retrospective; FNAB and CNB simultaneously; lesions with previous ndg.	CB: 20 G; FN: 25 G;	42 (36)*	48 (33)*	69 (51)*	21 (18)*
Sung et al. [22]	2012	South Korea	Retrospective; FNAB and CNB simultaneously	CN: 18 G; FN: 21, 23 and 25 G; automatic biopsy gun used	521	34	547	8
Park et al. [23]	2011	South Korea	Retrospective; lesions with previous ndg. FNAB	CN: 18 G, FN: no data; automatic biopsy gun used	73	69	53	1
Renshaw et al. [6]	2007	USA	Retrospective; CNB and FNAB simultaneously – lesions with previous ndg. FNAB and also as first choice	FN: 25, 23 and 21 G; CN: 18, 20, 21 G	265	112	310	67
Strauss et al. [24]	2007	USA	CNB and FNAB – lesions with previous ndg. FNAB	CN: 20 G; FN: 22, 25 G	22	59	43	38
Karstrup et al. [25]	2001	Denmark	Palpable lesions only; FNAB and CNB simultaneously;	CN: 18 G, automatic biopsy gun used; FN: 21 G	75	2	68	9

*Results for lesions with only one prior non-diagnostic biopsy were included

*FN* – fine needle, *CN* core needle, *diagn.* diagnostic results, *ndg.* non-diagnostic results

**Table 2:** Studies assessing the usefulness of core-needle biopsy (CNB) not included in the meta-analysis

Author	Year	Country	Design	Needles	FNAB – diagn.	FNAB – ndg.	CNB – diagn.	CNB – ndg.
Yeon et al. [26]	2013	South Korea	Retrospective; lesions with previous ndg. FNAB; no control group	CN: 18 G; FN: no data; automatic biopsy gun used	No data	No data	135	2
Khoo TK [31]	2008	USA	CNB and FNAB simultaneously compared with lesions that underwent FNAB only	No data	296	15	303*	37*
Zhang et al. [32]	2007	USA	Retrospective; CNB and FNAB simultaneously, in most cases after two ndg. FNABs	CN: 20, 22 G; FN: 25, 23 G	409	39	217*	8*
Mehrotra et al. [33]	2005	UK	Retrospective; US-guided CNB and freehand FNAB compared	CN: 20 G, automatic biopsy gun used; FN: 21 or 23 G	75	66	102	19
Harvey et al. [34]	2004	UK	Retrospective; CNB in random patients; FNAB partially without sonographic guidance	CN: 18 G; FN: 21–25 G;	159	107	69	10
Screaton et al. [35]	2002	UK	Retrospective; no control group; CNB – lesions with previous ndg. FNAB and also as first choice	CN: 16–18 G	No data	No data	199	10

*Summary data for simultaneous CNB and FNAB – without distinction of FNAB and CNB component

*FN* – fine needle, *CN* core needle, *FNAB* fine-needle aspiration biopsy, *diagn.* diagnostic results, *ndg.* non-diagnostic results

(Table 2)

We have also performed some analyses in subgroups.

**Fig. 3:**
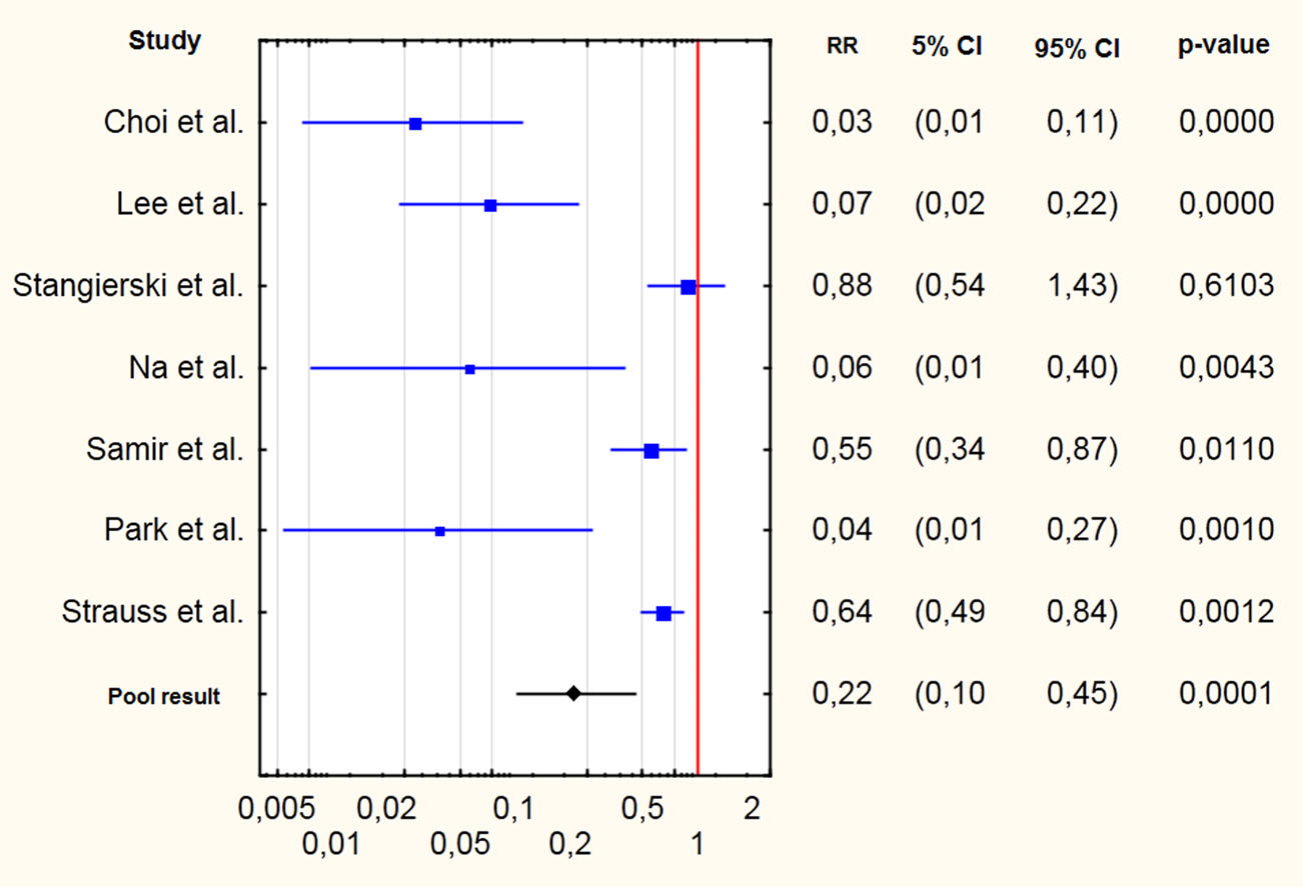
Cumulative forest plot for studies comparing risk ratios (RRs) of gaining non-diagnostic results with core-needle biopsy in comparison to fine-needle aspiration biopsy; with 95 % confidence intervals and p-values given in columns 2–4

Seven studies focused on lesions with one previous non-diagnostic result of FNAB [17–21, 23, 24]. The forest plot is shown on Fig. 3. The pooledRR of gaining a non-diagnostic result was 0.22 (95 % CI 0.10–0.45, p=0.0001). There is no evidence for publication bias (Kendall’s tau = −0.33, two-tailed p-value = 0.29). There was evidence of significant heterogeneity (Q =47.5, df=6, i^2^=87.37 %, p<0.0001).

Four studies from South Korea were performed with very similar methodology [17, 18, 20, 23]. Lesions with one previous non-diagnostic FNAB were included, in all studies the ACECUT system by TSK, Japan was used. For these studies the pooled RR was 0.05 (95 % CI 0.02–0.10, p<0.0001). ). There is no evidence for publication bias (Kendall’s tau = 0.0, two-tailed p-value = 1.0). There was no evidence of significant heterogeneity (Q =1.2, df=3, i^2^=0.0 %, p=0.76).

## Discussion

CNB yielded a significantly higher percentage of diagnostic results than FNAB in lesions with previous non-diagnostic results with FNAB. RR was 0.27, which means that the probability of gaining a non-diagnostic result was almost four times lower. However, the number of studies comparing the diagnostic efficacy of FNAB was rather low. We found 11 case-control studies on the topic. In addition, these studies differed with regard to the diameters of needles and design of the study (CNB as the first-line procedure or as a procedure performed after one or more non-diagnostic FNABs, prospective/retrospective character, simultaneous CNB and FNAB, or CNB and FNAB performed in distinct groups of subjects). Among these studies, nine had shown significantly higher diagnostic effectiveness of CNB, in one the difference was not significant [19], and in one FNAB had a significantly higher percentage of diagnostic results with borderline significance [25].

We also performed subgroup analyses. We found seven studies comparing FNAB and CNB in lesions with previous non-diagnostic results with FNAB; however, those studies were diverse in terms of methodology. Among those seven studies, four showed that CNB yielded incomparably higher diagnostic effectiveness – RR of non-diagnostic result <0.1 [17, 18, 20, 23]. According to two studies, CNB was significantly more effective; however, the result was less impressive than that of the four studies mentioned above [21, 24]. Another study did not reveal any advantage of CNB over repeated FNAB [19].

These discrepancies suggest that further studies are strongly recommended. One of the possible reasons could be different diameters of fine and core needles used in particular studies. Stangierski et al. [19] used 25 G fine and 22 G core needles, Samir et al. [21] used 25 and 20 G, respectively; in the study performed by Na et al. [20] 18 G needles were used for CNB and different types of needles for FNAB (21–25 G, proportion unknown), Lee et al. [18] used 18 G core needles, data about fine needles were not given. Similarly, Yeon et al. [26] reported very a high percentage of diagnostic results with CNB with 18 G needles in lesions after one non-diagnostic FNAB (over 98 %); however, this study was not included in the meta-analysis due to the lack of a control group.

Another possible reason for this heterogeneity could result from many variables, rather difficult to meta-analyse, such as experience of the radiologist/endocrinologist performing the biopsy, number of passes, equipment used, etc. Four studies performed using similar equipment (automatic biopsy guns from the same manufacturer) in patients with one previous non-diagnostic FNAB showed very homogenous results [17, 18, 20, 23]. This fact can suggest that equipment used and group of patients selected are the most important factors influencing the findings; homogeneity in these two areas resulted in very homogenous results. Pooled results of these studies were very impressive – the risk of gaining a non-diagnostic result was 20 times lower than in the case of FNAB. According to this data, automatic biopsy guns can be helpful in patients with non-diagnostic results with FNAB. However, the invasiveness of the procedure should also be taken into account.

Another aspect worth considering is a pain sensation among patients undergoing both kinds of procedures. The number of studies on this topic is limited. Reports performed by Stangierski et al. [19] and Nasrollah et al. [27] indicate CNB is slightly more painful than FNAB; however, it is tolerable for most patients. But it is worth keeping in mind that the difference in the diameter of core and fine needles in both studies was quite small – 21G versus 23G. In a study performed by Capri et al. [28], fine needles and large needles were used. The authors report no difference in pain sensation accompanying the two procedures; however, a small amount of anaesthesia was injected subcutaneously before the biopsy. According to the accessible data, it seems that the use of core needles is not accompanied by patient intolerance and a severe pain sensation.

The current study constitutes a large meta-analysis which aims to systematize this important topic. An interesting study on this issue was published by Trimboli et al. in 2014 [29]. These authors found and briefly described many previously published studies. However, it was systematic review so there was no quantitative synthesis of the results. There was also one conceptually similar meta-analysis published by Li et al. [30]. However, the authors of that study included a smaller number of studies, five, whereas 11 studies were included in the current study. Partially this difference can be explained by the fact that quite a few studies have been published recently and were not available for Li et al. [16–19]. A greater number of included studies allowed for additional analyses in subgroups (e.g. comparison of FNAB and CNB in lesions with one previous non-diagnostic FNAB). Finally, the meta-analysis published by Li et al. brought some confounding results: a visible, but nonsignificant difference in the diagnostic values of FNAB and CNB. Our study including more studies provides more definite, clinically important conclusions.

In conclusion, the CNB seems to be a valuable diagnostic technique yielding a higher proportion of diagnostic results than conventional FNAB. It is also significantly more effective in cases of nodules with prior non-diagnostic results with FNAB than repeated FNABs. However, further studies on the topic are required.
